# Environmental Influences on Food Behaviour

**DOI:** 10.3390/ijerph16152763

**Published:** 2019-08-02

**Authors:** Catherine Paquet

**Affiliations:** 1 Australian Centre for Precision Health, University of South Australia Cancer Research Institute, Adelaide, SA 5001, Australia; catherine.paquet@unisa.edu.au; 2 School of Health Sciences, University of South Australia, Adelaide, SA 5001, Australia

The ubiquitous presence of food cues in our modern environment is believed to contribute to the rising trends in overconsumption and associated obesity observed over the last few decades. This has led to the rapid development of a body of literature on how the food environment (the places where we purchase or consume food) promote healthy or unhealthy eating, obesity, and chronic diseases. The evidence emerging from this body of work has however failed to provide strong evidence supporting the health impact of the local food environment, with many studies reported null or counter-intuitive findings [1,2,3].

Although it is possible that the local food environment has only a negligible effect on food behaviors, the absence of clear relationships can also be attributed to limitations in the current body of research, especially in the way the food environment is conceptualized and measured. An example of such limitations is the heavy reliance on location data on food retailers that are classified as either ‘healthy’ or ‘unhealthy’ based on the retail category they belong to (e.g., fast food, corner stores, supermarkets) without consideration of the nutritional quality of the food available and promoted within each food outlet. Other limitations include a lack of attention to factors beyond physical proximity that may influence patronage such as psychological and social factors, and an emphasis on the food environment around the residence over other spaces that individuals are exposed to during their day.

This special issue on Environmental Influences on Food Behavior was conceived to address some of these limitations and aimed at advancing the research on environments shaping food-related decisions in terms of measurement and conceptualization of food environments and how, and under what circumstances, they influence specific purchasing and eating patterns across the life course. The special issue brings together a geographically and methodologically diverse collection of studies aiming to better understand the context in which we make everyday food decisions and the impact of this context on health-related outcomes. Some of the common themes and contributions emerging from the special issue are highlighted below.

## 1. Spatial Measures of Food Environment in Understudied Populations and Moderating Factors

Despite some recent progress, most of the food environment literature using proximity or density measures of food retailers remains focused on Western populations and urban settings. Two studies from the special issue explore how these relationships apply in relatively understudied contexts. The study by Zhang and Huang [4] for example, is one of the few studies investigating the local retail food environment in Asia. Their study, based on cross-sectional data on almost 2000 Hong Kong residents, suggests that infrequent fruit and vegetable consumption was more likely in individuals living in areas with the highest ratio of fast-food and convenience stores to grocery stores. As for other studies conducted in Western countries [1], a retail food environment index assessing the relative exposure of healthy to unhealthy food (or vice versa) seemed to be a more robust predictor of health-related outcomes than measures based on availability of specific types of food stores. Another unique study context is provided by the study conducted by Wycherley and colleagues [5] who investigated food access and diets in remote Indigenous communities in Australia. The authors used sales data from community stores to derive community-level food consumption estimates and observed that communities located further away from a neighboring store had a lower proportion of their energy intake coming from either discretionary foods or sugar-sweetened beverages. Although these results are mostly descriptive and would need to be replicated in a larger sample, they highlight the particular importance of community-level factors such as food sources within and outside the community for these remote communities. The study by Mackenbach and colleagues [6] also adds to the current body of cross-sectional studies investigating the relationship between the density of food outlets (fast food and general food retailers) around the residence and the odds of being obese, by investigating if, and how, this relationship varied according to the degree of self-control and financial strain experienced in a sample of almost 3000 Dutch adults. The results suggested that the relationship was mostly present in participants experiencing high financial strain or reporting low levels of self-control. Interestingly, a greater density of fast-food restaurants was associated with lower body mass index (BMI) in this population.

## 2. The Importance of Perceptions

It is recognized that *perceptions* of proximity, over and beyond the actual proximity, may also be an important driver of purchasing decisions. Two papers in this issue explore this idea further by investigating how perceptions of proximity influence food-related decision making and the factors that might influence these perceptions. Alves and colleagues [7] investigated parent’s perception of travel time to supermarkets and restaurants and reported that greater perceived travel time to restaurants (fast-food or full-service), but not supermarkets, was associated with a children’s dietary pattern consistent with less fast-food consumption and this effect was consistent across income levels. The concept of perceived distance was also investigated by Baldock and colleagues [8], who reported on the discrepancy between the perceived and objective distance to local fruit and vegetable retailers and the factors associated with these differences. The study revealed a range of factors were related to a greater likelihood of overestimating distances to the nearest fruit and vegetable retailer, including having a lower household income, poorer physical functioning, lower sense of community, more positive mental well-being and living in proximity to fruit and vegetable retailers.

## 3. Alternative Methods to Gain Insights into Food-Related Decisions

Although the above studies contribute to the existing literature, they remain mostly focused on traditional quantitative measures of the presence or density of food retailers within one’s local (residential) environment. Much insight can be gained into the context in which everyday food decisions are made by adopting alternative methodologies, as illustrated in three of the studies in this special issue. For instance, Sandín Vázquez and colleagues [9] adopted a qualitative approach and interviewed residents, neighborhood workers, and food traders to better capture social aspects related to food purchasing behaviors in a neighborhood of Madrid. Main key drivers of purchasing decisions emerging from the study were the importance of tradition and the trust and social relationships between customers and the food traders, the time scarcity associated with modern life, which drove younger residents towards supermarkets with greater ranges and convenience of food and longer opening hours, as well as the reduced purchasing power of individuals, which had a direct impact on purchasing decisions. This need for convenience also emerged as an important factor in the study by Riggsbee et al. [10], who used grounded visualization and story mapping informed by geographic information systems (GIS), focus group and photovoice data collection to study the food environment and behaviors of teenagers in the United States. The results indicated that healthy eating was supported at home, but busy schedules meant that convenience was often a key driver of food habits. The use of technology for menu planning, preparation and shopping was another theme that emerged from the study. Finally, Veatupu and colleagues [11] also used a new methodology, wearable cameras, to gain an in-depth understanding of the type of food consumed by 10–12 years children from Tonga and the context in which that food was obtained and consumed. The results highlighted that children obtained food—in particular, non-core foods—from a number of different sources including (but not limited to) other children, supermarkets, and convenience stores. The findings also demonstrated that children consumed food in a range of settings, including on the road, and purchased on average close to one non-core food product per day.

## 4. Insights into and from the Food Industry

The food environment is shaped by a number of actors from the food industry. The perspectives and practices of these various actors, as well as the factors shaping their practice, need to be considered to identify key leverage points for public health interventions. Three studies in the special issue attempt to do so by looking into marketing strategies used by retailers and seeking the views of key food industry actors on factors influencing the supply and demand for fruits and vegetables. For instance, in a scoping review of the marketing literature, Castro and colleagues [12] summarize evidence from 41 experimental studies conducted in real or simulated retail environment on factors influencing food purchase intentions or choices. The key factors identified from the review included shelf display, branding, nutrition labeling, food sampling, and price and price promotions, all factors that could be, or are already used, to promote healthy food choices. Many of these in-store factors are already recognized as being important aspects of the food environment, as demonstrated by the article by Horacek and colleagues [13] who developed and validated a practical tool for assessing the in-store food environment in convenience stores: The convenience store supportive healthy environment for life-promoting food (SHELF) audit tool. Finally, insights from the food industry were provided in the study by Gerritsen and colleagues [14] who used cognitive mapping interviewing to analyze information collected from a range of stakeholders (actors of the produce industry, food distribution, and retail sector, as well as governmental and non-governmental health organizations) to not only better understand the factors perceived to have contributed to a decline in fruit and vegetable intake in New Zealand children, but also identify potential solutions. Examples of barriers to fruit and vegetable consumption were related to limited household food budgets and high prices of fruit and vegetables, which were linked by some interviewees to extreme weather events and high supermarket margins. Other barriers included promotions and advertising of unhealthy food options, limited availability of fresh produce in some areas, lack of time and skills for food preparation, as well as limited supply due to loss of fertile land and government regulations. These results highlight the multiple factors contributing to shaping our food environment and the need to take a wider or systems perspective to develop effective and sustainable strategies to support healthy eating in communities.

In summary, the special issue sheds new light on the complexity of conceptualizing, measuring, and understanding the impact of the food environment on food decisions and health and on the importance of considering new methods and new perspectives to better understand, and intervene on, the context in which everyday food decisions are made. Whilst the progress reflected in this issue is encouraging, more research is still needed to more comprehensively capture environmental factors and food sources (including online sources) influencing food purchasing decisions in order to better inform the development of innovative interventions and policies to better support and promote healthier food habits.
